# Supplementary material for: Augmenting the Angiogenic Profile and Functionality of Cord Blood Endothelial Colony-Forming Cells by Indirect Priming with Bone-Marrow-Derived Mesenchymal Stromal Cells
Source: Biomedicines. 2023 May 5;11(5):1372. doi: 10.3390/biomedicines11051372 (PMC10216643; doi:10.3390/biomedicines11051372)
Supplement: Supplementary file 1 [file biomedicines-11-01372-s001.zip › biomedicines-2283504-supplementary.pdf]

Supplementary, Biomedicines, MDPI

CB-ECFC Phase Contrast

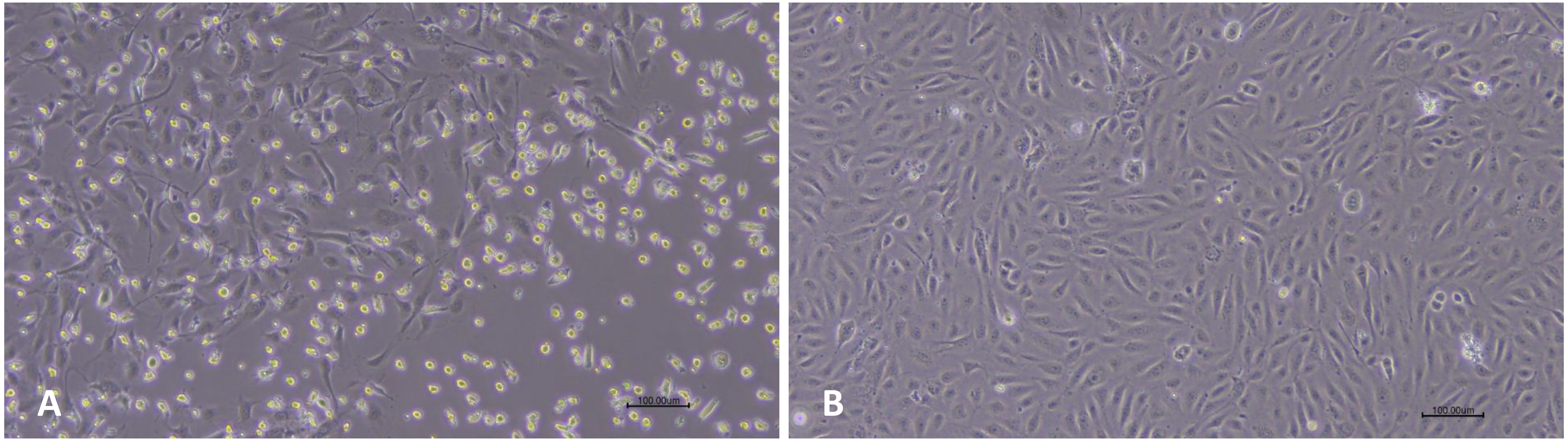

Figure S1.(A) depicts a typical ECFC colony isolated from umbilical cord blood on the day of appearance at 10x magnification; and **(B)** depicts ECFC culture at approximately 80% confluency with typical cobblestone morphology at 10x magnification. **Scale bar:100µm.**

Flow Cytometry of CB-ECFC

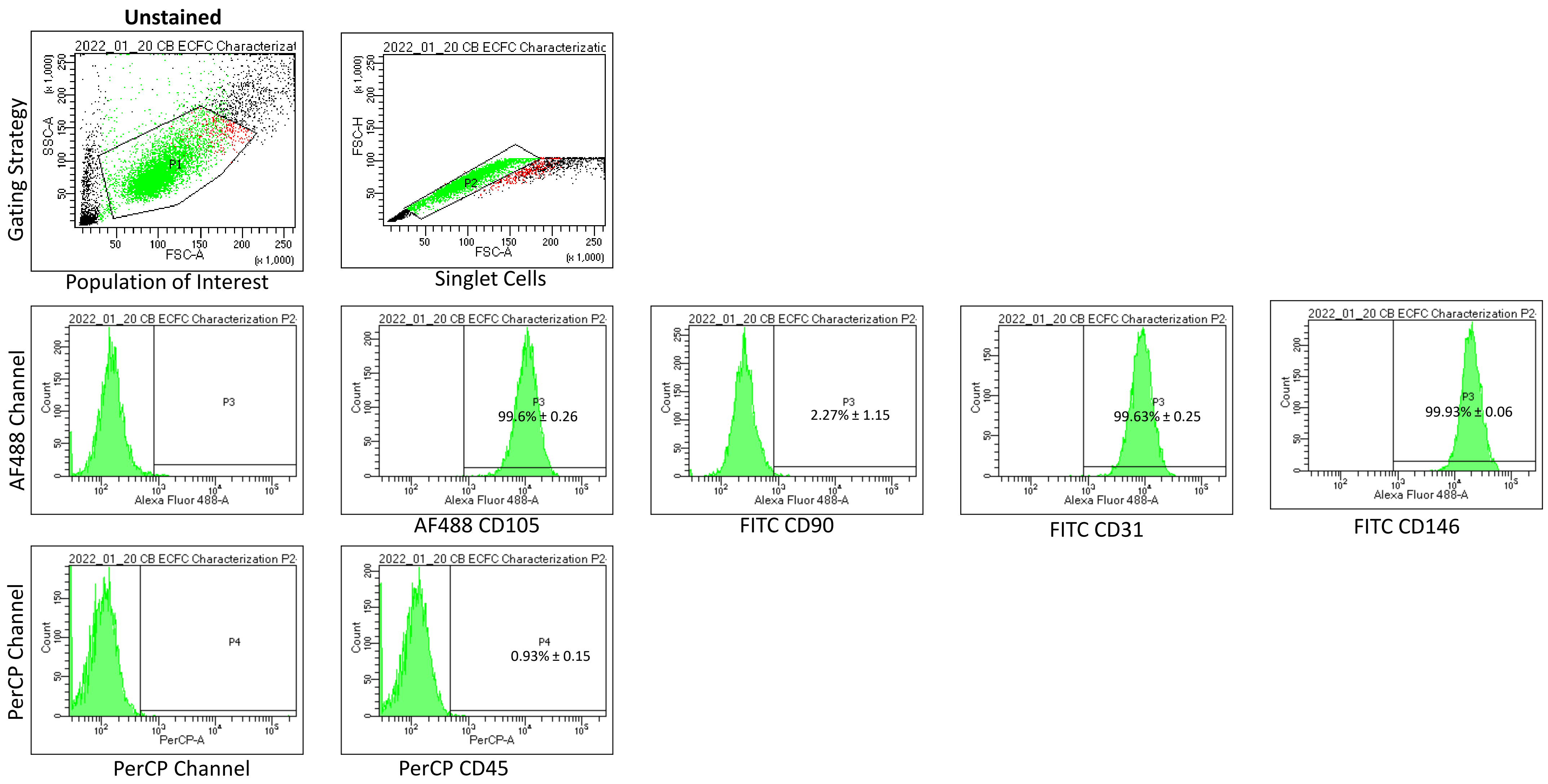

Contd.

Flow Cytometry of CB-ECFC

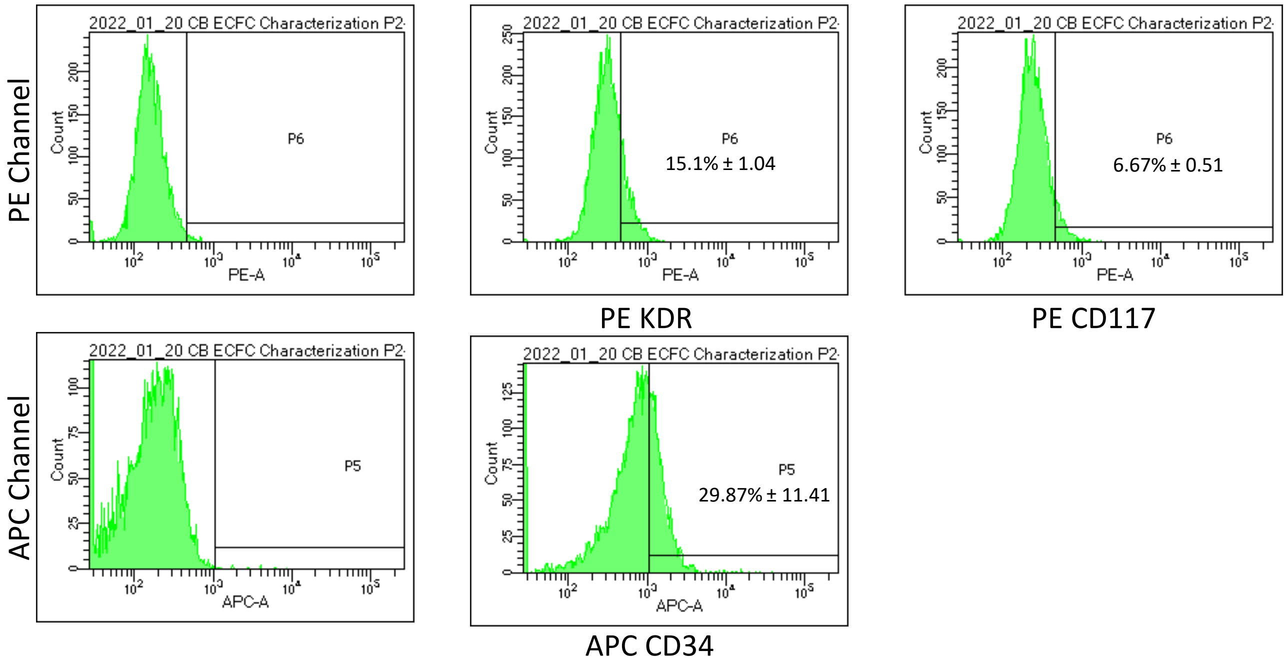

Figure S2. Histogram of immunophenotypic characterization of cord blood-derived ECFCs based on cell surface markers including endothelial markers (CD146, CD31, and KDR); progenitor markers (CD34, CD105, and CD117); leukocytic marker (CD45); and fibroblast marker (CD90). The left panel shows an unstained sample and the right panel shows the expression of specific cell surface markers. The scale represents a positive expression of markers with percent expression ± standard deviation. In the histogram, X-axis represents the fluorescence signal in the log scale and Y-axis represents the count of events.

KDR & CD34 Expression on CB-ECFC

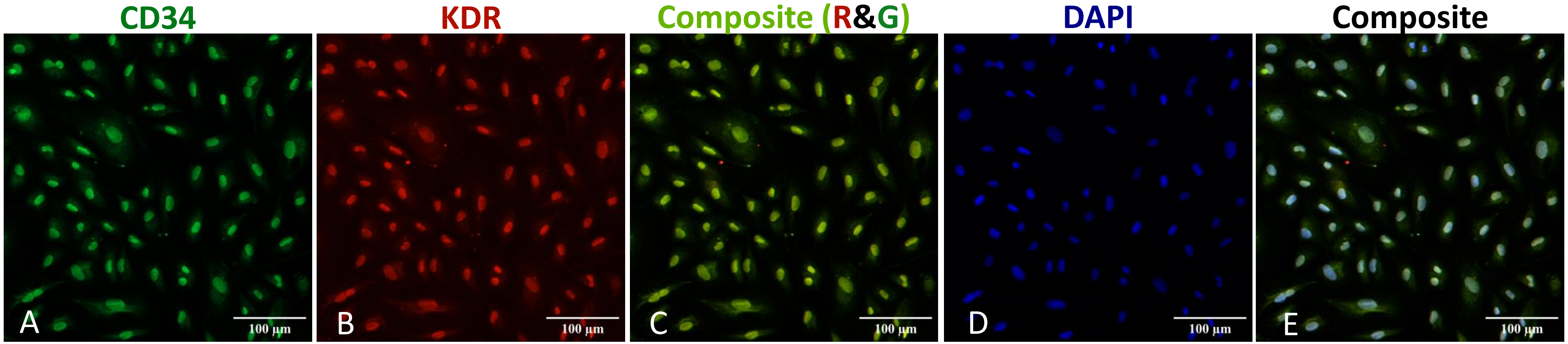

Figure S3. Confocal microscopy images depicting expression of progenitor marker and endothelial marker on umbilical cord blood-derived ECFCs. **(A)** Progenitor marker CD34 (Green); **(B)** Endothelial marker KDR (Red); **(C)** Composite image of CD34 and KDR; **(D)** DAPI (Blue) for nuclear stain; and **(E)** Composite image (RGB). All the images are at 20x magnification. **Scale bar: 100µm.**

AcLDL Uptake & Ulex Binding CB-ECFC

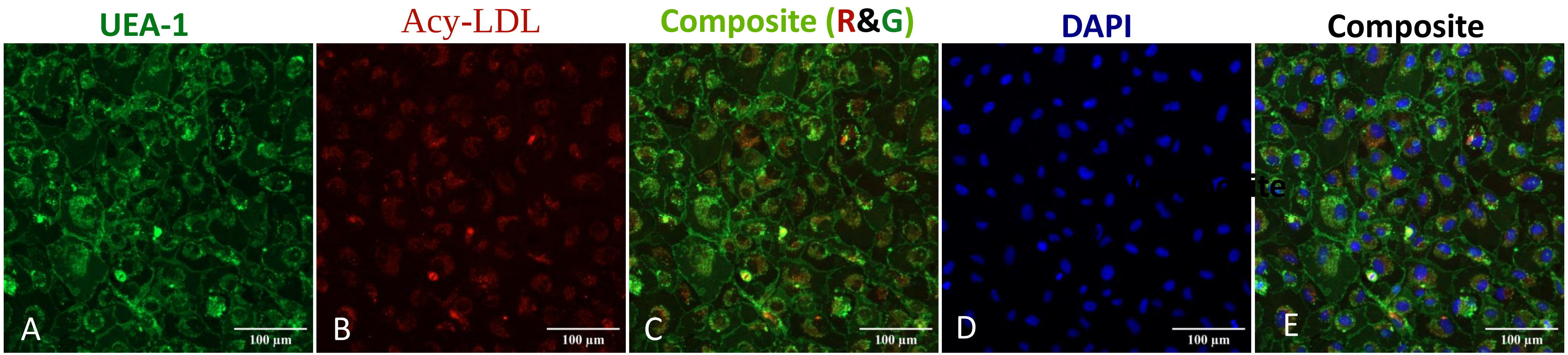

Figure S4. Confocal microscopy images for FITC-UEA-1 lectin binding and uptake of Dil-Acy LDL by ECFCs. **(A)** UEA-1 lectin binding; **(B)** Dil-Acy LDL uptake; **(C)** Composite image of UEA-1 lectin binding and Dil-Acy LDL uptake; **(D)** DAPI for nuclear staining; and **(E)** Composite image. All the images are at 20x magnification. **Scale bar: 100µm.**

BM-MSC Phase Contrast

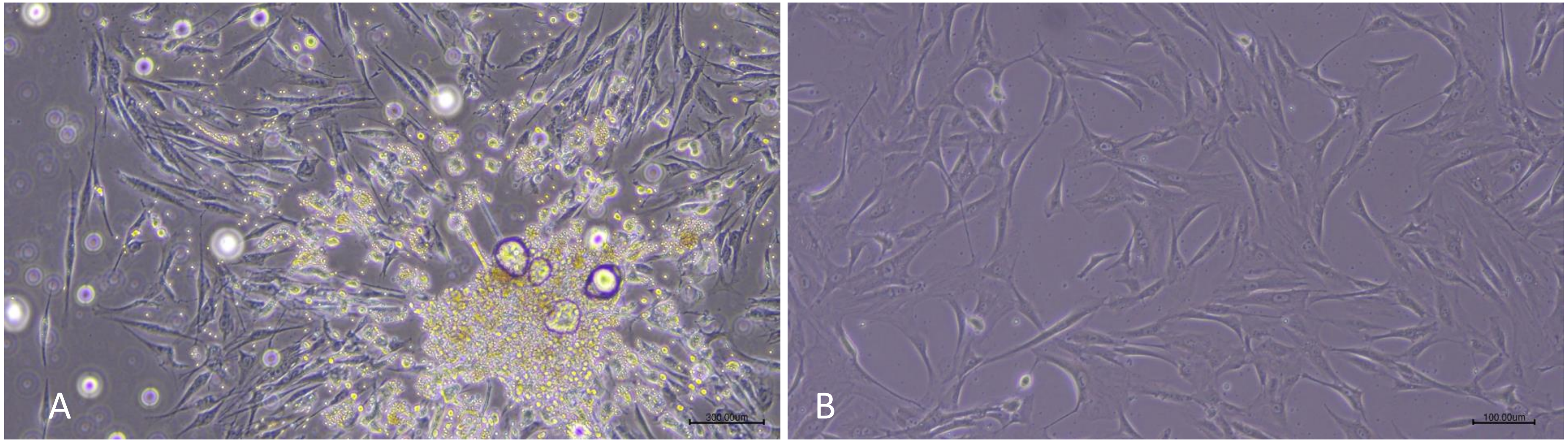

Figure S5. (A) depicts an MSC colony on the day of appearance isolated from human bone marrow at 10x magnification; and **(B)** depicts MSC culture at approximately 80% confluency with typical fibroblast morphology at 10x magnification. **Scale bar: 100µm.**

Flow Cytometry of BM-MSC

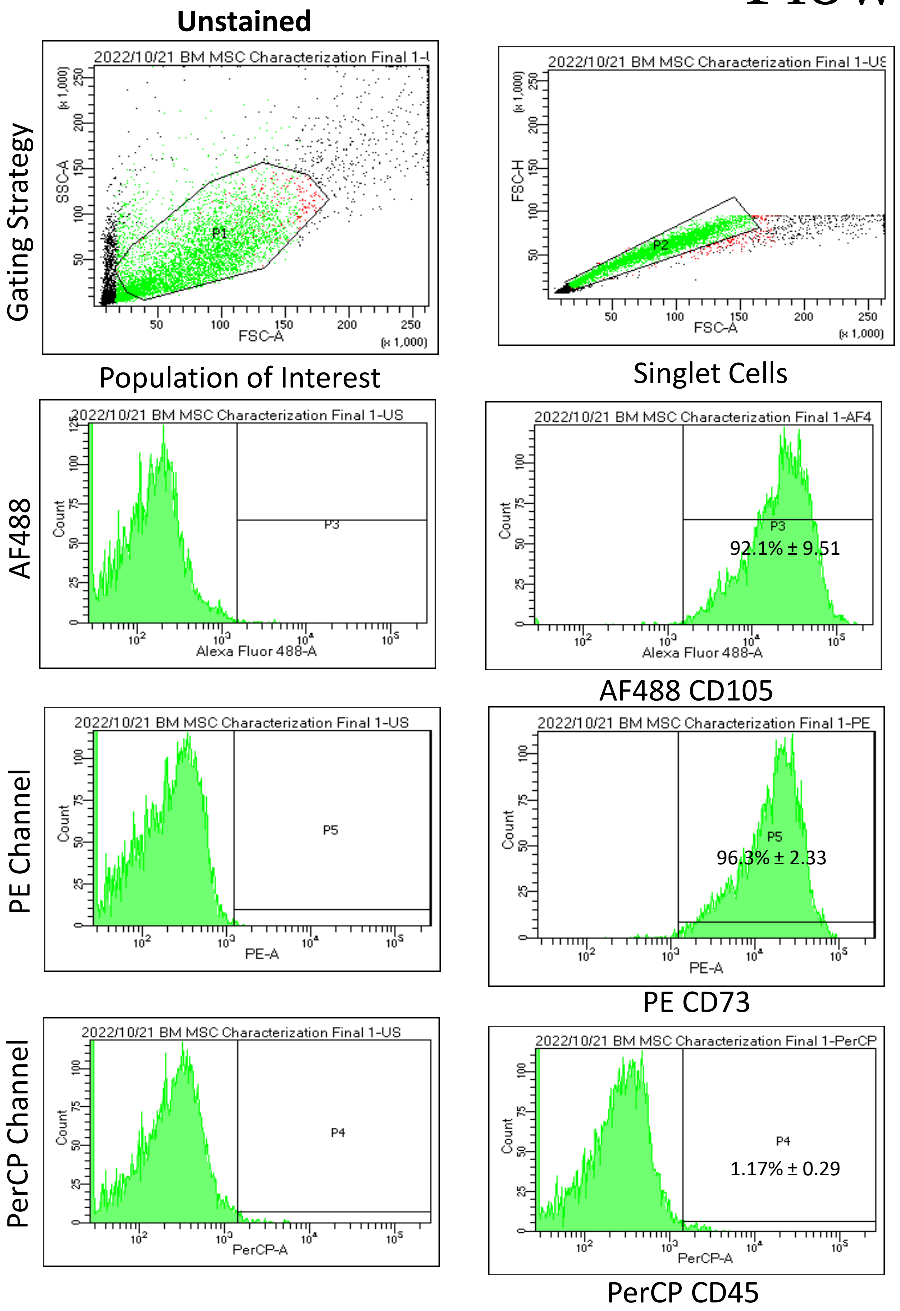

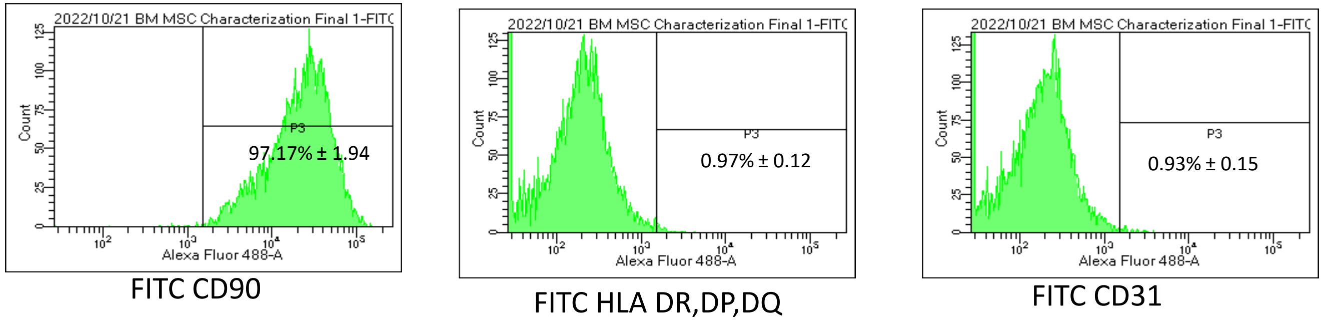

Figure S6. Histogram of immunophenotypic characterization of bone marrow-derived MSCs based on cell surface markers including mesenchymal and progenitor markers (CD90, CD73, and CD105); leukocytic marker (CD45); Immunogenicity marker (HLA-DR, DP, DQ); and endothelial markers (CD31). The left panel shows an unstained sample and the right panel shows the expression of specific cell surface markers. The scale represents a positive expression of markers with percent expression ± standard deviation. In the histogram, X-axis represents the fluorescence signal in the log scale and Y-axis represents the count of events.

Tri-lineage Differentiation

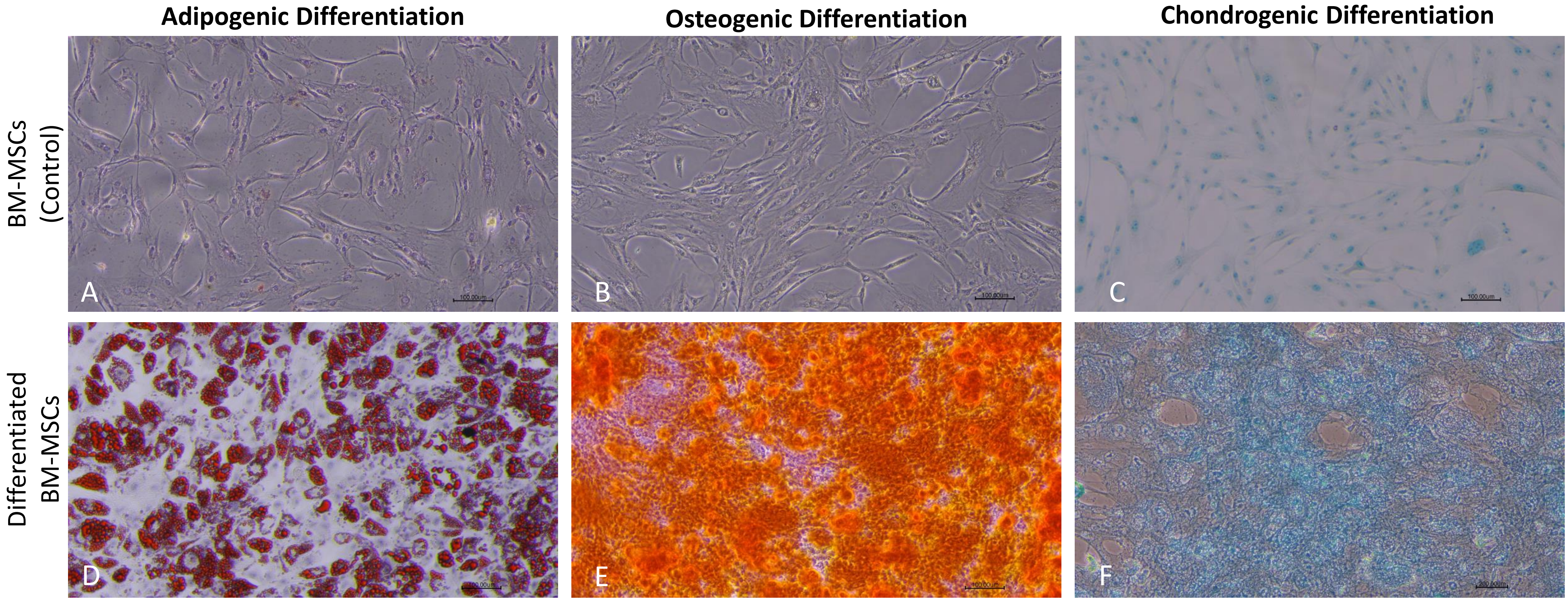

Figure S7. Trilineage differentiation potential of BM-MSCs. **(A)** Undifferentiated BM-MSCs stained with Oil Red O; **(B)** Undifferentiated BM-MSCs stained with Alizarin Red; **(C)** Undifferentiated BM-MSCs stained with Alcian Blue; **(D)** Differentiated BM-MSCs stained with Oil Red O depicting accumulation of lipid in Adipocytes; **(E)** Differentiated BM-MSCs stained with Alizarin Red depicting deposition of calcium by osteoblasts; and **(F)** Differentiated BM-MSCs stained with Alcian blue depicting formation of collagen by chondrocytes. All the images are at 10x magnification. **Scale bar: 100µm.**

Post-Sorting

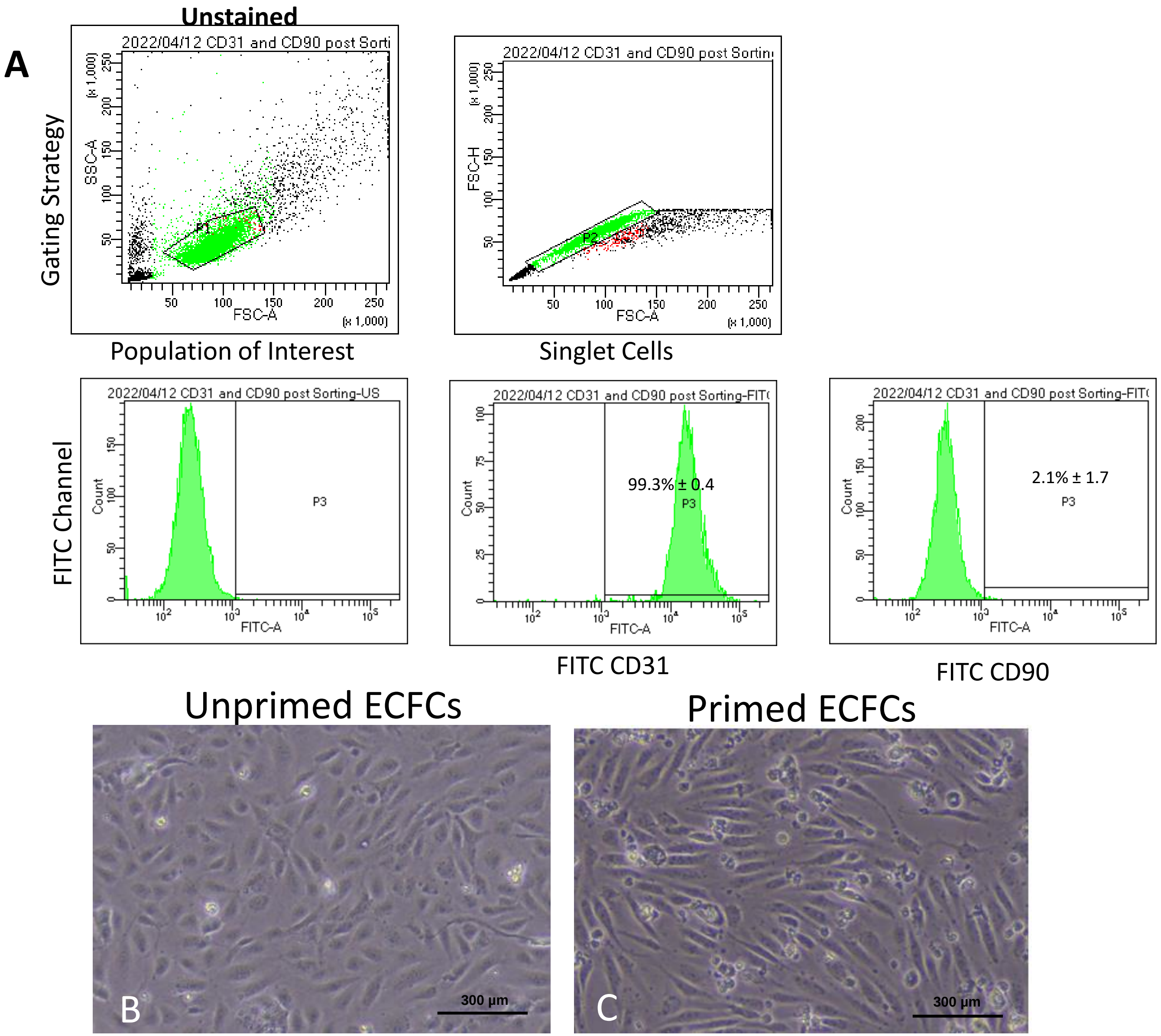

Figure S8. (A) Flow cytometric validation shows isolation of pure ECFC (positive for CD31 and negative for CD90) with minimal contamination of MSCs (Positive for CD90 and negative for CD31) after sorting through MACS. **Direct Priming of ECFCs with MSCs impart more fibroblast morphology to ECFCs (B)** Representative Images of Unprimed ECFCs exhibiting typical cobblestone morphology, whereas **(C)** Directly primed ECFCs exhibited more fibroblast-like morphology. All the images are at 10x magnification. **Scale bar: 300µm.**

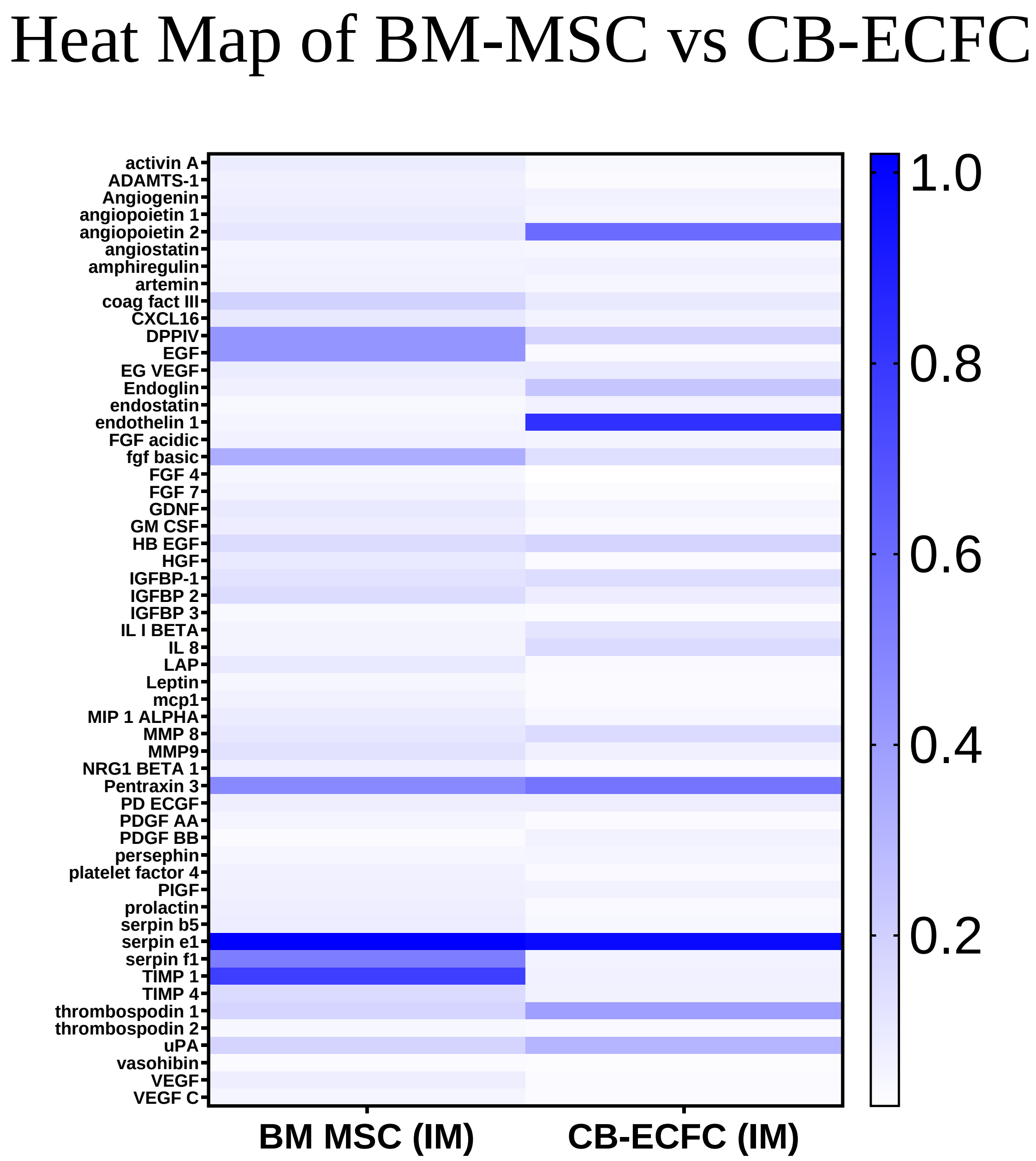

Figure S9. Differential protein expression of various angiogenesis-related proteins in BM-MSC(IM) and CB-ECFC (IM) displayed through a heat map.

Heat Map of all 4 comparison groups

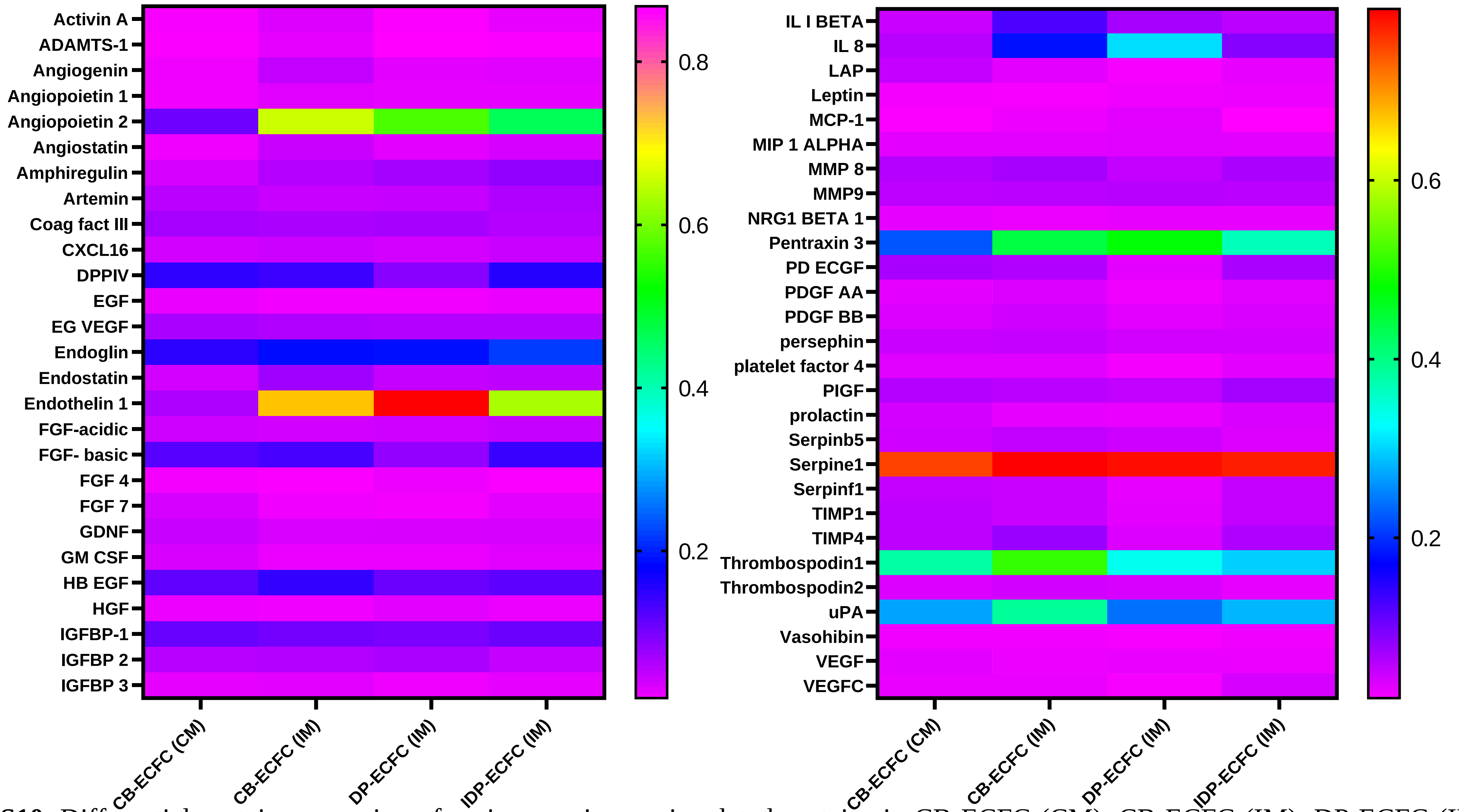

Figure S10. Differential protein expression of various angiogenesis-related proteins in CB-ECFC (CM), CB-ECFC (IM), DP-ECFC (IM), and IDP-ECFC (IM) displayed through a heat map.
